# Vaginal Douching in Cambodian Women: Its Prevalence and Association With Vaginal Candidiasis

**DOI:** 10.2188/jea.JE20081046

**Published:** 2010-01-05

**Authors:** Lon Say Heng, Hiroshi Yatsuya, Satoshi Morita, Junichi Sakamoto

**Affiliations:** 1 National Center for HIV/AIDS, Dermatology and STD, Ministry of Health, Phnom Penh, Cambodia; 2 Department of Young Leaders’ Program in Health Care Administration, Nagoya University Graduate School of Medicine, Nagoya, Japan; 3 Department of Public Health, Nagoya University Graduate School of Medicine, Nagoya, Japan; 4 Department of Biostatistics and Epidemiology, Yokohama City University Medical Center, Yokohama, Japan

**Keywords:** reproductive health, vaginal douching, vaginal candidiasis, Cambodia

## Abstract

**Background:**

We determined the prevalence of vaginal douching (cleansing of the vagina with liquid) in a sample of Cambodian women, and examined the associations of douching with genitourinary symptoms and infections, after controlling for potential confounding factors, including genitourinary symptoms and sociodemographic factors.

**Methods:**

A total of 451 adolescent and adult females aged 15 to 49 years who attended 17 maternal and child health (MCH) clinics in 7 provinces of Cambodia in 2001 were consecutively enrolled as a part of the Sexually Transmitted Infection Sentinel Survey. Sociodemographic factors, genitourinary symptoms, and frequency of douching were assessed by face-to-face interviews using a structured questionnaire. Vaginal infections were examined by using standard diagnostic procedures specific to each pathogen.

**Results:**

The proportion of participants who douched at least once a week was 76.7% (*n* = 346). Douching was significantly more prevalent in urban than in rural women (85.7%, *n* = 198 vs 67.3%, *n* = 148; *P* < 0.001). Frequency of douching was significantly associated with genitourinary symptoms, which were most prevalent in participants who douched from several times a week to once a day; genitourinary symptoms were less prevalent in those who douched more than once a day. Douching was significantly associated with vaginal candidiasis, but not with trichomoniasis or bacterial vaginosis, and this association persisted even after controlling for sociodemographic factors and genitourinary symptoms.

**Conclusions:**

Vaginal douching was very common among Cambodian women visiting MCH clinics. Further investigations are warranted to elucidate the reasons for douching. In addition, women should be informed that douching may endanger their reproductive health.

## INTRODUCTION

Vaginal douching is the process of washing or cleaning out the vagina with water or other fluid mixtures.^1^^,^^2^ Adverse health effects associated with vaginal douching have been widely reported,^1^^–^^5^ although uncertainty remains regarding causality, that is, whether douching causes disease or women douche in response to symptoms.^6^^,^^7^ It is generally recognized that women should be dissuaded from douching, given the preponderance of evidence supporting its potential harm,^1^^,^^2^ as compared to women who do not douche.^5^^,^^8^^,^^9^ Nevertheless, it is only recently that the potential health risk of vaginal douching has been documented, and public health messages to discourage women from douching have not been widely disseminated even in countries such as the United States, where the issue has been rather extensively studied.^10^^–^^12^ For example, the prevalence of vaginal douching was 22.4% in the US National Health and Nutrition Examination Survey, with significant differences between ethnic groups and rates as high as 50.2% among non-Hispanic blacks.^13^

In Cambodia, high prevalences of bacterial vaginosis (12%) and vaginal candidiasis (39%) have been reported among women who visited maternal and child health (MCH) clinics.^14^ Because these infections are related to low birth weight, infant and maternal mortality, and other negative reproductive health outcomes,^15^^–^^17^ determining the host factors that are associated with these infections in Cambodian women would be especially beneficial in reducing Cambodia’s heavy disease burden. Based on our understanding of the association between douching and these infections, we first determined the prevalence of douching and its relationship with sociodemographic factors. Such information should prove useful in developing culturally relevant interventions against douching. In addition, if the prevalence of douching is indeed high, and the practice is causally related to adverse health outcomes, douching interventions would have a significantly greater public health impact than would just targeting interventions focused on high-risk individuals, such as commercial sex workers.^18^^,^^19^

In order to identify independent associations between douching and infections, we examined the associations among douching, vaginal symptoms, and infections, after controlling for the confounding effects of vaginal symptoms and sociodemographic factors.

## METHODS

### Study area and participants

The study was carried out in 7 selected provinces as a part of the Sexually Transmitted Infection (STI) Sentinel Survey, which was conducted by the National Center for HIV/AIDS, Dermatology and STI (NCHADS) from December 2000 through March 2001, and specifically targeted low-risk women. Other parts of the STI survey included men and direct (brothel-based) sex workers as target populations. To recruit study participants, 17 MCH clinics affiliated with 8 urban health centers and 9 rural health centers were selected. Eligible participants were adolescent and adult females aged 15 to 49 years who visited MCH clinics affiliated with health centers for antenatal care, family planning, or consultations regarding their children. These participants were considered to be a group of females that closely resembled the Cambodian general population. Women who are at higher risk of infection and transmission, such as female sex workers, do not usually visit health centers; instead, they go to special free clinics that provide regular medical checkups at least once a month under the aegis of local authorities.

The participants were consecutively recruited until the estimated sample size was reached. The total sample size was determined in the original survey on the assumption that the prevalence of STI was 10%, and that the 90% confidence interval of the prevalence was within 2.4 percentage points of 10% (ie, from 7.6% to 12.4%). This calculation yielded the number of 423, which was rounded up to 450 to compensate for lost samples, should sampling problems occur. Of the 451 subjects, 48.8% (*n* = 220) were recruited in rural areas and 51.2% (*n* = 231) in urban areas.

The study was approved by both the Cambodian Ministry of Health Ethical Review Committee and the Protection of Human Subjects Committee of Family Health International for all procedures, including the protocol, consent forms, and questionnaires.

### Questionnaire-based interview

Using a behavioral surveillance questionnaire, the interviewer posed questions about age, marital status, and place of residence. Physicians then made inquiries about issues related to lower genital symptoms, including vaginal discharge, vaginal itching, and dysuria, as well as the practice of vaginal douching. The frequency of vaginal douching was categorized as: more than once a day, once a day, a few times a week, once a month, and never. For our analyses, we combined once a day and a few times a week into 1 category, and once a month and never into another. Douching once a month was considered to be equivalent to never douching, since vaginal flora may revert to normal levels during a 1-month period. Because douching more than once a day could lead to the alleviation of symptoms, this extreme category was dealt with separately in the analyses. The use of interviews to assess douching was considered to be accurate because speaking about douching practices is common among Cambodian women, and the questions were posed by female physicians^16^ and female interviewers, all of whom had undergone thorough training.

### Sample collection and laboratory methods

Pelvic and speculum examinations were performed after the face-to-face interviews in a setting that ensured each participant’s privacy. Vaginal swabbing was performed systematically to collect vaginal fluid from the posterior fornix using 2 cotton swabs while avoiding cervical secretions.^15^ One swab was prepared for a microscope slide or air-dried for Gram staining to examine for bacterial vaginosis (BV). The second swab was inoculated into the culture medium to examine for trichomoniasis. The procedure was as follows: a cotton swab with vaginal fluid was inoculated into the upper chamber of the pouch of an InPouch TV Test.^20^ The pouches were sealed and transported to the laboratory at the end of each day. They were then examined at the national laboratory in Phnom Penh City on arrival, incubated at 37 °C, and examined by microscopy at 12, 24, and 48 hours post-incubation to determine the morphology and motility of the protozoa. BV was determined based on a Gram-stain examination of vaginal fluid using a standardized 0 to 10 point scoring system based on 3 morphotypes.^21^ Candida albicans was diagnosed microscopically based on the presence of characteristic budding yeast cells and hyphae.

### Statistical analysis

Categorical variables were tested by the chi-square or Fisher exact tests. Multivariate logistic regression analysis was used to calculate odds ratios (ORs) and their 95% confidence intervals (95% CIs), while controlling for potential confounding variables. A 2-tailed *P* value less than 0.05 was considered statistically significant. The analyses were performed using SPSS 12.0 for Windows.

## RESULTS

The mean age of the participants was 26.5 years (standard deviation, 6.5 years). More than 95% of participants were married (Table 1), and over 70% were housewives or self-employed farm workers.

**Table 1.** Sociodemographic characteristics of participants, by frequency of vaginal douching

Variables	Frequency of douching			*P*-value

	Never/once a month or less	Several times a week/once a day	More than once a day	
	*n* = 105	*n* = 50	*n* = 296	
Age group (years)				
<19	10 (9.5)	3 (6.0)	28 (9.5)	0.56
20–24	38 (36.2)	16 (32.0)	120 (40.5)	
25–29	22 (21.0)	12 (24.0)	71 (24.0)	
≥30	35 (33.3)	19 (38.0)	77 (26.0)	

Present marital status				
Married	103 (98.1)	50 (100.0)	290 (98.0)	0.60
Other	2 (1.9)	0 (0.0)	6 (2.0)	

Occupational status				
Housewife/farmer	83 (79.0)	41 (82.0)	218 (73.6)	0.30
Others	22 (21.0)	9 (18.0)	78 (26.4)	

Place of residence				
Urban	33 (31.4)	34 (68.0)	164 (55.4)	<0.001
Rural	72 (68.6)	16 (32.0)	132 (44.6)	

Numbers in parentheses are percentages.

*P* values were determined by using the chi-square test or Fisher exact test.

Of the 451 participants, 296 (65.6%) douched more than once a day, and 50 (11.1%) did so at least once a week but less than once a day. Only water was used for douching by 306 (88.4%), while others used commercial products such as Betadine (10% povidone-iodine) solution and Sanoformine (mixture of anhydrous copper sulfate and sodium fluoride).

Douching was not associated with either the age or marital status of the participants. There tended to be fewer housewives among those who douched more than once a day, as compared to participants reporting other douching frequencies. Those residing in urban areas were likely to douche more often than participants in rural areas (*P* < 0.001). Adjustments for other factors presented in Table 1 did not alter the association between douching and place of residence.

Vaginal symptoms were significantly associated with the frequency of douching (Table 2). The symptoms were most prevalent in participants who douched from several times a week up to once a day, followed by participants who douched more than once a day. Although vaginal symptoms were more prevalent in participants with vaginal infections, none of these associations was statistically significant (*P* > 0.05), although the associations of dysuria with trichomoniasis (*P* = 0.061) and itching with BV (*P* = 0.063) tended toward significance (Table 3).

**Table 2.** Association between frequency of vaginal douching and genitourinary symptoms in Cambodian women

Frequency of douching	Vaginal discharge		Dysuria		Vaginal itching	
		
	Prevalence *n* (%)	*P*	Prevalence *n* (%)	*P*	Prevalence *n* (%)	*P*
Never/once a month or less	41 (39.0)	0.001	13 (12.4)	0.015	27 (25.7)	0.003
Several times a week/once a day	34 (68.0)		16 (32.0)		27 (54.0)	
More than once a day	163 (55.1)		58 (19.6)		107 (36.1)	

^a^*P* values were determined by using the chi-square test.

**Table 3.** Associations between vaginal infections and the presence of genitourinary symptoms in Cambodian women

Symptoms	Trichomoniasis		

	positive *n* (%)	negative *n* (%)	*P*^a^
vaginal discharge	7 (53.8)	231 (52.6)	0.78
dysuria	5 (41.7)	82 (18.7)	0.061
itching	6 (50.0)	155 (35.3)	0.36

	Bacterial vaginosis		
	
vaginal discharge	29 (55.8)	206 (52.2)	0.66
dysuria	14 (29.6)	72 (18.2)	0.14
itching	25 (48.1)	134 (33.9)	0.063

	Vaginal candidiasis		
	
vaginal discharge	95 (54.3)	143 (51.8)	0.63
dysuria	39 (22.3)	48 (17.4)	0.22
itching	66 (37.7)	95 (34.4)	0.48

^a^*P* values were determined by using the Fisher exact test.

Vaginal candidiasis was dose-dependently associated with the frequency of douching (Table 4). Multivariate adjustment for age, place of residence, marital and occupational status, and symptoms of vaginal itching, dysuria, and vaginal discharge did not materially alter this association.

**Table 4.** Results of logistic regression analyses of the associations between the frequency of vaginal douching and vaginal infections in Cambodian women

Frequency of douching	Trichomoniasis		

	Prevalence *n* (%)	OR (95% CI)	OR2 (95% CI)
Never/once a month or less	2 (1.9)	1 (reference)	1 (reference)
Several times a week/once a day	3 (6.0)	3.29 (0.53–20.33)	1.28 (0.17–9.86)
More than once a day	7 (2.4)	1.25 (0.26–6.10)	0.91 (0.17–4.90)

	Bacterial vaginosis		
	
Never/once a month or less	11 (10.5)	1 (reference)	1 (reference)
Several times a week/once a day	9 (18.0)	1.86 (0.72–4.82)	1.16 (0.40–3.27)
More than once a day	32 (10.8)	1.04 (0.50–2.14)	0.85 (0.40–1.84)

	Vaginal candidiasis		
	
Never/once a month or less	32 (30.5)	1 (reference)	1 (reference)
Several times a week/once a day	17 (34.0)	1.18 (0.57–2.40)	1.14 (0.53–2.47)
More than once a day	126 (42.6)	1.69^a^ (1.05–2.72)	1.82^b^ (1.10–3.00)

OR, odds ratio; CI, confidence interval.

OR2: Adjusted for age, place of residence, marital and occupational status, and vaginal symptoms (vaginal discharge, dysuria, or vaginal itching).

^a^*P* value = 0.030; ^b^*P* value = 0.019.

## DISCUSSION

This study conducted in Cambodia is the first to confirm that the practice of vaginal douching is very common among Cambodian adolescent and adult females sampled in 17 MCH clinics in 7 provinces, with two-thirds of participants reporting douching more than once a day. The prevalence documented in the present study is even higher than those reported elsewhere.^12^^,^^22^^–^^24^ In fact, a previous review stated that most women do not usually douche every day.^1^^,^^2^ Further studies will be needed to confirm whether our finding is true for Cambodian women only, or whether the present sample might have included more participants with higher rates of douching. However, we believe that the sample is generally representative of Cambodian women.

In our study, the majority of participants used water alone (88.4%) for douching. This proportion was similar to that among Turkish women (70%). In Western countries, however, commercial products seem to be used more often,^10^^,^^23^^–^^26^ though such products were not commonly (11.6%) used by the participants interviewed in our present study. This may be due to the fact that such products are uncommon in the Cambodian marketplace and more expensive. We previously performed an unstructured interview survey at 8 family health clinics, where the physician interviewed at least 3 women each about their douching methods. The most common method was to use their fingers, that is, women first wrap cotton wool around their fingertips and dip them into a bowl or basin filled with warm water, to which they sometimes add salt, alum, lemon juice, or commercial products. They then insert their fingers into their vagina and wipe it clean. This procedure is usually repeated 2 or 3 times for each douching session. The next common method is to use a large plastic pot filled with warm water. Women sit on the pot and then insert their fingers into their vagina to cleanse it. Another method utilizes devices for spraying warm water into the vagina, such as a syringe, a plastic tube connected to a commercial squeeze bottle, or home-made gravity feed drip bottle that is designed to be hung on a hook at approximately the height of an adult. Douching was performed at home or in a private toilet. Women learn about douching and its techniques mostly from their mothers, and sometimes from their friends and neighbors.

At this writing, we cannot explain the high prevalence of vaginal douching among Cambodian women. Because urban women had a significantly higher prevalence of douching than did rural women, there may be cultural, socioeconomic, or environmental factors that favor douching in urban areas, such as access to private toilets or a clean water supply or misconceptions about hygiene. There is clearly a great need for additional studies to carefully collect information on the reasons underlying douching practice. In any case, the most important implication for the public health of Cambodian women is that health care workers must initiate organized activities to disseminate accurate knowledge of vaginal hygiene in varying settings, such as MCH clinics, communities, and high schools, in order to immediately discourage women from douching.

**Figure 1.**
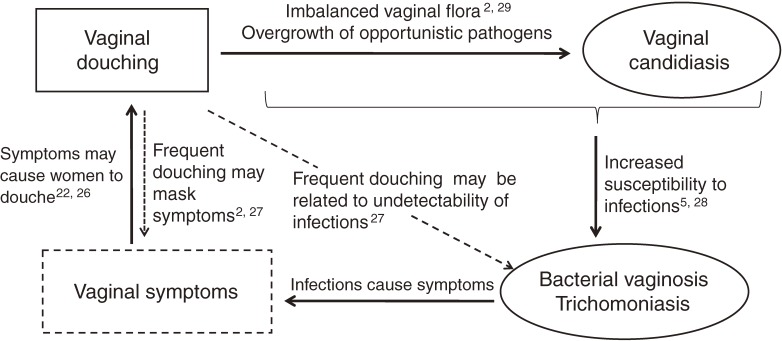
Relationships among douching, genitourinary symptoms, vaginal candidiasis, bacterial vaginosis, and trichomoniasis.

Douching was associated with complaints of vaginal discharge, vaginal itching, and dysuria, a finding that is consistent with other studies.^22^^,^^26^ However, it was puzzling to discover that these symptoms were more prevalent in women who douched several times a week than in those who douched more frequently. It is possible that frequent douching, as often as once or more a day, might have masked such symptoms. Although these symptoms were found to be associated with BV and trichomoniasis, we did not observe any association between douching and these infections (Figure 1).^2^^,^^27^ If douching was a host response to the symptoms caused by these infections, it would be natural to observe an association between the infections and douching. However, frequent douching itself might have made the infections difficult to detect. Alternatively, it is possible that a woman who douches more than once a day had douched before their visit, which may have made it difficult to detect an infection.

Douching was associated with candidiasis in a dose-dependent manner independent of confounders. This is consistent with a study of Australian women with symptoms of abnormal vaginal discharge or odor, which found that only candidiasis was associated with douching, while BV was associated with indicators of high-risk sexual behavior.^28^ Although causality cannot be judged from a cross-sectional study, our finding that douching was dose-dependently associated with vaginal candidiasis, and that multivariate adjustments did not materially alter this association, suggests that douching is a cause of that condition. Moreover, frequent douching is reported to change vaginal pH and microflora concentrations by removing normal flora and permitting the overgrowth of opportunistic pathogens,^2^^,^^29^ a finding that also supports inference causative role for douching. Furthermore, in one clinical study, valvovaginal candidiasis was associated with diabetes and recent antibiotic use, suggesting the importance of host factors.^30^ Of course, there is the possibility that other unmeasured confounders may explain the association between douching and candidiasis. One such factor could be the existence of multiple sexual partners; however, the present sample included only low-risk women, ie, almost all were married, and no women had reported an extramarital relationship in the past year.

Interestingly, in another study of women with already imbalanced flora, douching was associated with BV.^5^ This report suggests the importance of maintaining vaginal flora, thus supporting recommendations to avoid douching as routine hygiene, since BV may be related to a serious reproductive outcome such as spontaneous abortion or preterm delivery.^15^ Douching could also induce a vicious cycle of douching, candidiasis, BV, and symptoms, that is, symptoms caused by BV lead to douching, and imbalanced flora due to douching lead to candidiasis. Such a state would be related in turn to further susceptibility to BV.

There were several limitations in the present study. First, the participants were not questioned as to the reasons why they douched. Although the participants might have considered douching as routine feminine hygiene,^10^^–^^12^^,^^22^^,^^23^^,^^26^ it is essential to know whether women had douched or not in response to vaginal symptoms, in order to make a causal inference.^2^^,^^6^ This limitation together with the cross-sectional nature of the present study make it impossible to determine the temporality of any association. However, we speculate that candidiasis, which was dose-dependently associated with douching independent of symptoms, could have been caused by douching. Second, the study used data collected at MCH clinics affiliated with health centers. Although almost all the women in the present sample were married, and women seeking antenatal care are generally considered at low risk of STI,^31^ those visiting family planning clinics could reportedly include women at higher risk.^32^ However, because family planning/birth spacing programs have been implemented in an attempt to reduce poverty in Cambodia, and married Cambodian women are unlikely to have extramarital sex, the present sample can be considered to consist of low-risk women. At the same time, there may be other reasons why women visited these clinics, and women with unmeasured characteristics that were associated with douching or symptoms might have been over-represented in the present sample. Generalizability of the findings observed in the present study should thus be approached with caution. Community-based studies enrolling all women living in a given area may be warranted to confirm the high prevalence of douching in Cambodian women. Third, use of antibiotics and the presence of other underlying medical conditions were not assessed. Diabetes mellitus and contraceptive use, for example, have been reported to be risk factors for candidiasis,^30^ and the presence of factors such as these might have biased the present results. Future studies should collect these data and control for their confounding effects. Finally, the cross-sectional design precludes any investigation of the temporal relation between infection and douching: there was no way to determine whether douching was the cause or the effect of vaginal infection. Future studies should directly address whether frequent douching is associated with deterioration of vaginal flora, colonization of opportunistic pathogens (such as candida species) and/or subsequent acquisition of BV or trichomoniasis; they should also investigate whether frequent douching is beneficial for symptomatic women with apparent vaginitis or vaginosis. Public health agencies should be made aware of the importance of this problem and its potential impact on maternal and child health, and should be encouraged to support future studies.

In conclusion, we found that vaginal douching was very common among women visiting MCH clinics in Cambodia, and confirmed its association with valvovaginal candidiasis, but not with BV or trichomoniasis. Although causality was not definitively established, women should be informed that vaginal douching may carry a risk of vaginal candidiasis and endanger their reproductive health.
